# High Frequency of Haplotype HLA-DQ7 in Celiac Disease Patients from South Italy: Retrospective Evaluation of 5,535 Subjects at Risk of Celiac Disease

**DOI:** 10.1371/journal.pone.0138324

**Published:** 2015-09-23

**Authors:** Nadia Tinto, Arturo Cola, Chiara Piscopo, Marina Capuano, Martina Galatola, Luigi Greco, Lucia Sacchetti

**Affiliations:** 1 Department of Molecular Medicine and Medical Biotechnology, University of Naples “Federico II”, Naples, Italy; 2 CEINGE–Advanced Biotechnology, s. c. a r. l., Naples, Italy; 3 Department of Translational Medical Sciences, Section of Pediatrics, University of Naples “Federico II”, Naples, Italy; New York State Dept. Health, UNITED STATES

## Abstract

**Background:**

Celiac disease (CD) has a strong genetic component mainly due to HLA DQ2/DQ8 encoding genes. However, a minority of CD patients are DQ2/DQ8-negative. To address this issue, we retrospectively characterized HLA haplotypes in 5,535 subjects at risk of CD (either relatives of CD patients or subjects with CD-like symptoms) referred to our center during a 10-year period.

**Methods:**

We identified loci DQA1/DQB1/DRB1 by sequence-specific oligonucleotide-PCR and sequence-specific primer-PCR; anti-transglutaminase IgA/IgG and anti-endomysium IgA by ELISA and indirect immunofluorescence, respectively.

**Results:**

We diagnosed CD in 666/5,535 individuals, 4.2% of whom were DQ2/DQ8-negative. Interestingly, DQ7 was one of the most abundant haplotypes in all CD patients and significantly more frequent in DQ2/DQ8-negative (38%) than in DQ2/DQ8-positive CD patients (24%) (*p*<0.05).

**Conclusion:**

Our data lend support to the concept that DQ7 represents an additive or independent CD risk haplotype with respect to DQ2/DQ8 haplotypes but this finding should be verified in other large CD populations.

## Introduction

Celiac disease (CD) is an immune-mediated enteropathy triggered by gluten ingestion that may occur at any age in genetically predisposed individuals, and affects approximately 1% of the general population in Europe [1]. The strong genetic component of CD [2] is mainly due to HLA class II genes that encode the DQ2 and DQ8 molecules. Most (95–99%) CD patients, irrespective of age, carry these molecules that account for about 40% of the disease heritability [3,4], whereas other CD-associated genetic factors contribute little to the disease risk [5–7]. DQ2 exists in two highly homologous variants, DQ2.5 encoded by alleles DQA1*05/DQB1*02 and DQ2.2 encoded by alleles DQA1*02/DQB1*02; DQ2.2 is usually considered to entail a lower CD risk than DQ2.5 [8,9]. The presence of HLA-DQ2/DQ8 is necessary but not sufficient for the disease development. In fact, these molecules are also present in 30–40% of unaffected Caucasian subjects, but their absence is very rarely associated with a diagnosis of CD [1].

Given its high negative predictive value, HLA molecular typing is widely used to predict CD risk, particularly among relatives of CD patients [10,11], rather than to diagnose CD.

Recently, the European Society of Gastroenterology, Hepatology and Pediatric Nutrition (ESPGHAN) recommended HLA-typing to reinforce CD diagnosis and so avoid small intestinal biopsy in children and adolescents with gastrointestinal symptoms, IgA-tTG levels greater than 10 times the upper reference limit and EMA positivity [12]. However, a minority of DQ2/DQ8-negative CD patients, in a variable percentage depending on geographical area, develops the disease [13].

The above considerations prompted us to investigate the CD-associated HLA haplotypes in a cohort of 5,535 individuals at risk of CD (either relatives of CD patients or subjects with CD-like symptoms) from south Italy, enrolled over a period of 10 years (2003–2013), to identify common and uncommon HLA haplotypes associated with the disease in our geographical area.

## Materials and Methods

The retrospective analysis of HLA molecular typing was performed in 5,535 subjects at risk of CD referred to our Department of Laboratory Medicine of the University of Naples/Center of Advanced Biotechnology (CEINGE) of Naples, Italy, in a time window of ten years (2003–2013). A total of 1,785 subjects presented CD-like symptoms (1,254 ≤18 years and 531 >18 years) and 3,750 subjects were relatives of patients affected by CD (1,805 ≤18 years and 1,945 >18 years). Written informed consent to the study was obtained both from the adult enrolled subjects and from a parent or legal guardian of the enrolled children. The study was approved by the Ethics Committee of University “Federico II” of Naples and was conducted according to the Helsinki II declaration.

We studied loci DQA1, DQB1 and DRB1 using both sequence specific oligonucleotide-PCR screening (Dynal Biotech Ltd, Bromborough, UK) and the sequence specific primer-PCR (DQ-CD Typing Plus kit, BioDiagene, Palermo, Italy or HISTO TYPE Astra Formedic s.r.l. Milano, Italy) for confirmation, when necessary. Based on the type and number of the detected HLA alleles, we established the haplotypes and genotypes in each subject. The haplotypes assessed were: DQ2.5 = presence of DQA1*05/DQB1*02 (DRB1*03) alleles; DQ2.2 = presence of DQA1*02-DQB1*02 (DRB1*07) alleles; DQ2.3 = DQA1*03-DQB1*02 (DRB1*04/09/11) alleles; DQ8 = presence of DQA1*03-DQB1*0302 (DRB1*04) alleles; DQ7 = presence of DQB1*0301/0304 (DRB1*11/12/X) alleles. The DQ9, DQ4, DQ5 and DQ6 haplotypes were assigned if DQB1*0303, DQB1*04, DQB1*05 or DQB1*06 alleles were present, respectively.

Anti-transglutaminase (tTG) IgA, or IgG in subjects with IgA deficiency, were tested by ELISA using human recombinant tTG as antigen (DIA Medix Corp., Miami, FL, USA). Total serum IgA was evaluated by a nephelometric assay (BN ProSpec System; Behring, Marburg Germany). Anti-endomysium IgA levels were measured by indirect immunofluorescence on rhesus monkey esophagus substrate (Eurospital, Trieste, Italy) in tTG IgA-positive subjects to exclude false positive results.

In the presence of CD-associated antibodies and depending on the patient’s age, CD was diagnosed in subjects with CD-like intestinal lesions at biopsy according to the Marsh classification [14] and ESPGHAN criteria [12].

Genotype and haplotype frequencies were reported as absolute value and in percentages. The statistical significance of differences between groups were evaluated by χ2 test and by binomial logistic regression analysis, *p* values <0.05 were considered significant. Statistical analysis was conducted with the PASW package for Windows (v18; SPSS Inc Headquarters Chicago IL, USA).

## Results and Discussion

**Fig 1:**
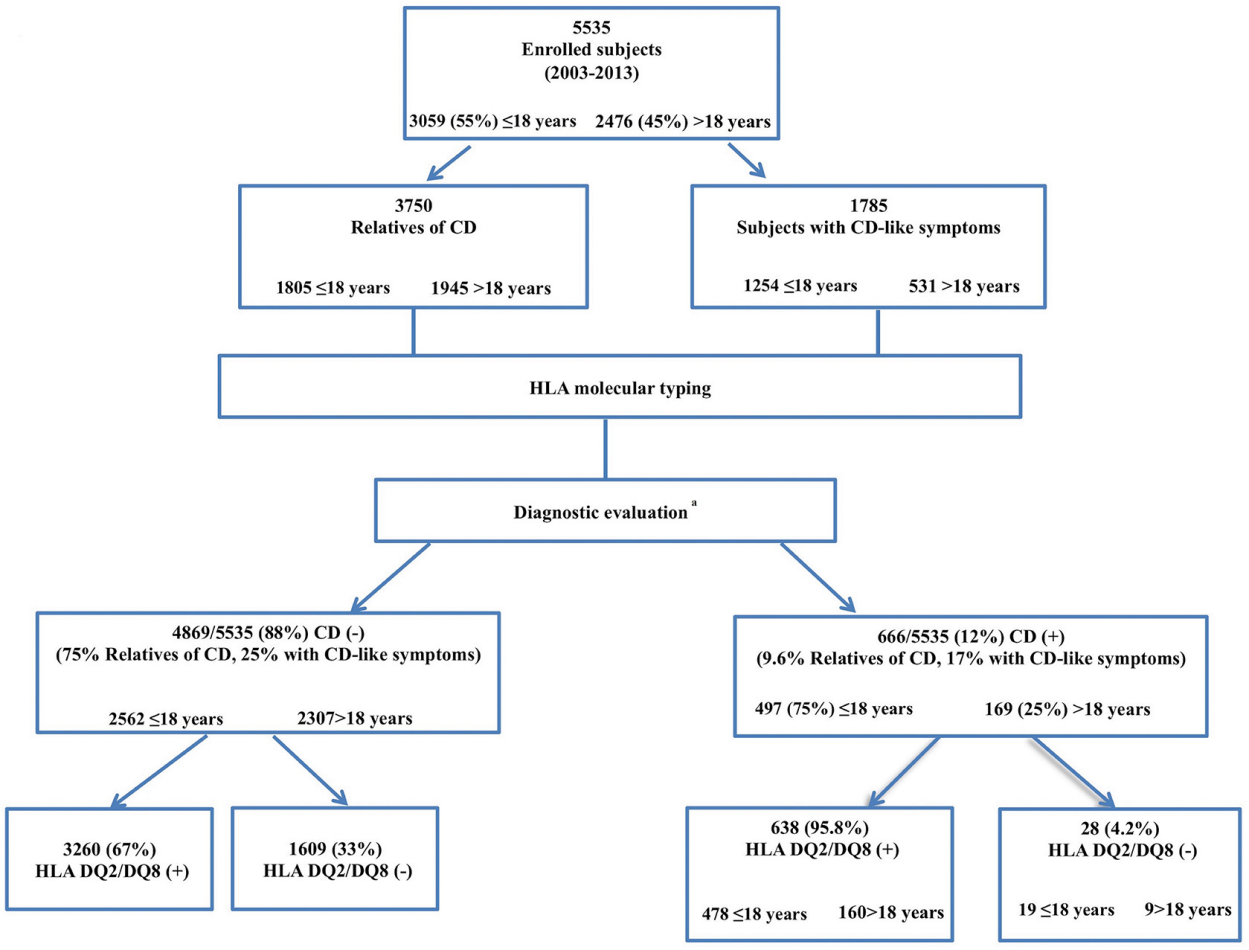
Flow-chart of the subjects at risk of CD from south Italy. Characteristics, age and presence/absence of HLA-DQ2/DQ8 in a population at risk of CD (relatives of CD- and with CD-like symptoms subjects) attending the Department of Laboratory Medicine of the University of Naples Federico II/CEINGE-Center of Advanced Biotechnology (Naples, Italy) between 2003 and 2013.

Among the 5,535 subjects at risk of CD enrolled in this study (3,750 relatives of CD patients and 1,785 with CD-like symptoms subjects), 3,059 were aged < 18 years and 2,476 were between 19 and 82 years of age (Fig 1). Celiac disease was diagnosed in 666/5,535 (12%) individuals; 75% of them (497 CD cases) were <18 years old (Fig 1). The prevalence of CD in the overall population was 9.6% in relatives of CD patients and 17% among subjects with CD-like symptoms, which is in agreement with previous data, namely, from 4% to 17% in CD relatives [11,15,16] and from 12% to 50%, in symptomatic subjects [15].

The prevalence of CD was higher (2.38:1) in children [497/3,059 (16.24%)] than in adults [169/2476 (6.8%)] (p<0.001), which tallies with previous Italian data (1.57:1) [17]. It was also higher in females than in males *(p*<0.001), the female/male ratio being 1.8–2.7 depending on the patients’ age (data not shown), which also coincides with previous reports (1.5–2.0) [15,18]. The HLA genotype distribution, after normalization for age and family history differed between males and females (Table 1A).

**Table 1:** Frequencies of HLA-DQ genotypes (A) and haplotypes (B) detected in 666 CD patients from south Italy.

**A)**			
**HLA-DQ GENOTYPES** ^**a**^	**M**	**F**	**TOT**
	n (%)	n (%)	n (%)
**DQ2 and/or DQ8 (+)**	**220 (33.0)**	**418 (62.8)**	**638 (95.8)**
**Double dose of DQ2 and/or DQ8**			
DQ2/DQ2	58 (25)^b^	82 (18.9)	140 (21.0)
DQ8/DQ8	2 (0.9)	0	2 (0.3)
DQ2/DQ8	16 (6.9)	26 (6.0)	42 (6.0)
**Single dose of DQ2 and/or DQ8**			
DQ2/DQX^c^	123 (53.1)	285 (65.6)^b^	408 (61.5)
DQ8/DQX^c^	21 (9.0)	25 (5.8)	46 (7.0)
**DQ2 and DQ8 (-)**	**12 (5.1)**	**16 (3.7)**	**28 (4.2)**
B)			
**HLA-DQ HAPLOTYPES**	**Entire CD cohort**	**DQ2/DQ8 (+) CD patients**	**DQ2/DQ8 (-) CD patients**
	**n (%)**	**n (%)**	**n (%)**
DQ2.5	371 (28.0)	371 (29.0)	**-**
DQ2.2	356 (27.0)	356 (28.0)	**-**
DQ7	324 (24.0)	303 (24.0)	21 (38.0)^d^
DQ5	108 (8.0)	95 (7.4)	13 (23.0)^d^
DQ8	92 (7.0)	92 (7.2)	-
DQ6	61 (4.6)	48 (3.7)	13 (23.0)^d^
DQ9	12 (0.9)	5 (0.4)	7 (13.0)^d^
DQ4	4 (0.3)	2 (0.1)	2 (3.0)
DQ2.3	3 (0.2)	3 (0.2)	-
Chromosomes	1332	1276	56

CD, celiac disease

^a^Genotypes were based on the presence of the following haplotypes: DQ2.5 = DQA1*05-DQB1*02 (DRB1*03) alleles; DQ2.2 = DQA1*02-DQB1*02 (DRB1*07) alleles; DQ2.3 = DQA1*03-DQB1*02 (DRB1*04/09/11) alleles; DQ8 = DQA1*03-DQB1*0302 (DRB1*04) alleles

^b^Statistically significant differences, *p*<0.001 at χ^2^ test between males (M) and females (F)

^c^DQX refers to: DQ7 = DQB1*0301 (DRB1*11/12/X) alleles; DQ4, DQ5, DQ6 and DQ9, were assigned if DQB1*04, DQB1*05, DQB1*06 and DQB1*0303 alleles were present, respectively

^d^Statistically significant differences, *p*<0.05 at χ^2^ test, between DQ2/DQ8 (+) and DQ2/DQ8 (-) CD patients

In our CD population, 95.8% (638/666 patients) were DQ2- and/or DQ8-positive (Table 1A). The DQ2 CD risk molecule was more frequent in a double dose in males than in females 25.0% and18.9%, *p* = 0.011) and less frequent in a single dose in males than in females (53.1% and 65.6%, *p* = 0.001). The DQ8 CD risk molecule was present in 0.3% and 7% of cases in a double or in a single dose, respectively, whereas DQ2/DQ8 molecules were present together in 6.0% of cases, irrespective of gender. In agreement with previous data [19], we found a slightly higher albeit not statistically significant (*p* = 0.47) percentage of HLA-DQ2/DQ8-negative CD patients in males (5.1%) than in females (3.7%) (Table 1A). Among DQ2 genotypes containing two CD risk molecules, DQ2.5/DQ2.2 was the most frequent (97 patients, 14%), whereas genotypes DQ2.2/DQX, DQ2.5/DQX and DQ8/DQX containing one CD risk molecule occurred in 218 (33%), 187 (28%) and 46 (7%) patients, respectively, in agreement with previous data (data not shown) [10,11]. Further characterization of the DQX haplotype revealed that DQ7 was the most frequent HLA haplotype (38%) in DQ2/DQ8-negative CD patients, and the third most frequent HLA haplotype (24%) in the entire CD cohort, irrespective of gender, family history and age (Table 1B).

Notably, our group of 4,869 unaffected subjects showed higher frequencies of the DQ2/DQ8 and DQ7 haplotypes (Fig 1 and Table 2), as expected being constituted by relatives of CD patients (consequently they had a similar genetic background as their relatives affected by CD) and by subjects with gastrointestinal symptoms (in whom high DQ2/DQ8 frequencies were previously described) [20].

**Table 2:** Frequencies of HLA-DQ genotypes (A) and haplotypes (B) detected in unaffected subjects (n = 4869) from south Italy.

A)			
**HLA-DQ GENOTYPES** ^**a**^	**CD-relatives**	**with CD-like symptoms**	**TOTAL**
	**n (%)**	**n (%)**	**n (%)**
**DQ2 and/or DQ8 (+)**	**2591 (71.0)** ^**b**^	**669 (55.0)**	**3260 (67.0)**
**Double dose of DQ2 and/or DQ8**			
DQ2/DQ2	374 (10.2)^**b**^	81 (6.7)	455 (9.4)
DQ8/DQ8	5 (0.1)	0	5 (0.1)
DQ2/DQ8	146 (4.0)	38 (1.4)	184 (3.8)
**Single dose of DQ2 and/or DQ8**			
DQ2/DQX^c^	1793 (48.9)^**b**^	461 (38.1)	2254 (46.3)
DQ8/DQX^c^	273 (7.4)	89 (7.4)	362 (7.4)
**DQ2 and DQ8 (-)**	**1071 (29.0)**	**538 (45.0)** ^**b**^	**1609 (33.0)**
B)			
**HLA-DQ HAPLOTYPES**	**Unaffected cohort**	**DQ2/DQ8 (+) unaffected subjects**	**DQ2/DQ8 (-) unaffected subjects**
	**n (%)**	**n (%)**	**n (%)**
DQ7	3009 (31.0)	1276 (19.6)	1612 (50.0)^d^
DQ2.2	1837 (19.0)	1837 (28.0)	-
DQ2.5	1510 (16.0)	1510 (23.0)	-
DQ5	1596 (16.4)	604 (9.2)	897 (28.0)^d^
DQ6	890 (9.1)	337 (5.1)	499 (15.5)^d^
DQ8	556 (6.0)	556 (8.5)	-
DQ9	218 (2.2)	91 (1.3)	145 (4.5)
DQ4	119 (1.2)	54 (0.8)	65 (2.0)
DQ2.3	3 (0.03)	3 (0.04)	-
Chromosomes	9738	6520	3218

CD, celiac disease

^a^Genotypes were based on the presence of the following haplotypes: DQ2.5 = DQA1*05-DQB1*02 (DRB1*03) alleles; DQ2.2 = DQA1*02-DQB1*02 (DRB1*07) alleles; DQ2.3 = DQA1*03-DQB1*02 (DRB1*04/09/11) alleles; DQ8 = DQA1*03-DQB1*0302 (DRB1*04) alleles

^b^Statistically significant differences between CD-relatives (n = 3662) and with CD-like symptoms (n = 1207) subjects; *p*<0.001 at χ^2^ test

^c^DQX refers to: DQ7 = DQB1*0301 (DRB1*11/12/X) alleles; DQ4, DQ5, DQ6 and DQ9, if DQB1*04, DQB1*05, DQB1*06 and DQB1*0303 alleles were present, respectively

^d^Statistically significant differences, *p*<0.001 at χ^2^ test, between DQ2/DQ8 (+) and DQ2/DQ8 (-) unaffected subjects.

Furthermore, the DQ7 haplotype was statistically more frequent in DQ2/DQ8-negative than in DQ2/DQ8-positive CD patients (38.0% *vs* 24.0%, *p* = 0.04) as well as in DQ2/DQ8-negative versus DQ2/DQ8-positive unaffected subjects (50.0% and 19.6%, respectively, *p*<0.001) (Tables 1B and 2B). Other haplotypes that differed statistically (*p*<0.05) between DQ2/DQ8-negative and DQ2/DQ8-positive CD patients were DQ5 (23% *vs* 7.4%), DQ6 (23% *vs* 3.7%) and DQ9 (13% *vs* 0.4%).

In our cohort, 4.2% of CD patients were DQ2/DQ8-negative (DQX/DQX), which is lower than the percent of DQ2/DQ8-negative CD patients (about 6%) previously reported in a large European CD population [13]. This discordance reflects the fact that the latter study was performed before DQ2.2 was identified as a CD-predisposing molecule [9], and hence was not considered a CD risk molecule. Furthermore, our results are in line with a higher prevalence of DQ2/DQ8-negative CD patients in south Europe than in north Europe [13].

The weight of the DQ7 (DQA05-DQB1*0301) haplotype in CD risk has been previously reported only in the presence of the DQ2.2 (DQA1*02-DQB1*0202) haplotype and was implicated in the production of the DQ2.5 molecule in trans [8].

Very recently a support to our descriptive data was given by Bergseng et al [21]. In fact, these authors demonstrated in lymphoblastoid cell lines and by relative quantitative proteomics that HLA-DQ2.5,-DQ2.2 and-DQ7.5 molecules have different specificity requirements for peptide binding and consequently distinct risks for celiac disease [21].

In conclusion, our results obtained in a large Italian cohort of children and adult CD patients lend support to the concept that DQ7 represents an additive or independent CD risk haplotype with respect to DQ2/DQ8 haplotypes. Moreover, our data questions the negative predictive value generally attributed to the absence of HLA-DQ2/DQ8 molecules in subjects at risk of CD. In fact, based on our results a diagnosis of CD should not be ruled out *a priori* in HLA-DQ2/DQ8-negative individuals carrying the HLA-DQ7 molecule, but this finding should be verified in other large CD populations.
